# The UK Tobacco Industry Interference Index 2023: A methodological approach

**DOI:** 10.18332/tpc/207097

**Published:** 2025-09-18

**Authors:** Tom Gatehouse, Karin Silver, Mary Assunta, Raouf Alebshehy

**Affiliations:** 1 Tobacco Control Research Group, Department of Health, University of Bath, Bath, United Kingdom; 2 Global Center for Good Governance in Tobacco Control, Bangkok, Thailand; 3 Southeast Asia Tobacco Control Alliance, Bangkok, Thailand

**Keywords:** tobacco industry interference, WHO FCTC Article 5.3, methodological approach, United Kingdom, global tobacco industry interference index

## Abstract

The UK Tobacco Industry Interference Index (UKTI) is part of the Global Tobacco Industry Interference Index (GTI). Based on a survey methodology developed by the Southeast Asia Tobacco Control Alliance (SEATCA), and published by the Global Center for Good Governance in Tobacco Control (GGTC), the GTI is a global survey on how governments respond to tobacco industry interference and to what extent they protect their public health policies from the commercial and vested interests of the tobacco industry, as required under the World Health Organization Framework Convention on Tobacco Control (WHO FCTC). Since the first GTI in 2019, the UKTI has been compiled by the Tobacco Control Research Group (TCRG) at the University of Bath. The latest UKTI, the fourth in the series, was published in November 2023. Monitoring, investigating and reporting on industry interference is complex, with large volumes of publicly available information found in multiple locations and formats. These include government sources such as lobbying registers, Hansard reports and registers of interests; tobacco industry sources such as company reports, websites and press releases; and external media including the industry and retail press. TCRG has implemented some innovative methods using a wide range of tools and resources, including open-source intelligence techniques (OSINT) and freedom of information requests (FOIs). These methods are outlined and critically assessed in this article. In doing so, we hope that lessons learned in the UK may be relevant to monitoring of tobacco industry interference elsewhere.

## INTRODUCTION

The UK Tobacco Industry Interference Index (UKTI) is part of the Global Tobacco Industry Interference Index (GTI)^1^, a global survey assessing to what extent governments protect their public health policies from the commercial and vested interests of the tobacco industry, as required by Article 5.3 of the World Health Organization Framework Convention on Tobacco Control (WHO FCTC) and the guidelines published subsequently to assist Parties in its implementation^2,3^.

The UK has participated in all four editions of the GTI to date, with the four UK reports having been compiled by the Tobacco Control Research Group (TCRG) at the University of Bath^4^.

Countries are awarded a score and ranked, the aim being to allow comparison across countries. The higher the score, the higher the level of tobacco industry interference. Prior to the UKTI 2023, the UK had been ranked no lower than fourth in the GTI. However, the UKTI 2023 recorded a sharp increase in the score and the UK fell from third to 21st place.

This article aims to outline the methods used to compile the 2023 UKTI, while providing some examples of sources used.

## METHODOLOGICAL APPROACH

The survey methodology was developed by the Southeast Asia Tobacco Control Alliance (SEATCA) and is based on the guidelines for implementation of Article 5.3 of the WHO FCTC^5^. Governments are assessed according to 20 indicators, grouped under seven key themes. This methodology provides the basic framework for the report, which in-country researchers complete.

A discussion of the SEATCA methodology is beyond the scope of this study; here our focus is how we compiled the 2023 report for the UK, using the tools, techniques and sources of information available to us.

In the UKTI, ‘government’ is defined broadly, capturing not only the UK government and devolved administrations (of Scotland, Wales, and Northern Ireland), but also the wider public sector, including the civil service, local authorities, opposition political parties and backbench Members of Parliament (MPs).

In the Article 5.3 guidelines, it is stated that ‘Parties should interact with the tobacco industry only when and to the extent strictly necessary to enable them to effectively regulate the tobacco industry and tobacco products’^3^. Interactions to which this does not apply, or which are not transparent, are considered for inclusion in the report.

This includes interactions with third-party organizations with known tobacco industry links, e.g. where an organization has tobacco industry members or a proven history of accepting tobacco industry funding. For reasons of capacity, while some relevant incidents relating to e-cigarette retailers and manufacturers were included – particularly where there are proven links to the tobacco industry – in general the e-cigarette sector was not monitored during the production of the UKTI 2023.

The period covered by the UKTI 2023 is from April 2021 to March 2023. However, the report also includes information on incidents which took place in the four years prior (April 2017 – March 2021), which were not covered in the previous reports. We also consulted the earlier reports to identify instances of industry interference which were still active or ongoing. A limited number of relevant incidents which occurred after March 2023 were included in the report to provide further detail, though they were not considered when calculating the score. Other instances of tobacco industry interference falling outside the timeframe have been recorded, with the aim of including them in the UKTI 2025.

A scoping review was conducted of the academic literature and publicly available evidence. This included websites of the UK government and devolved administrations, which provided a wide range of sources including public consultations, policy reviews, records of meetings, ministerial agendas, press releases, calls for evidence, official advice and others. UK parliament websites also provided much useful data including information on lobbying, transcripts of parliamentary debates, MPs’ and Lords’ registers of interest, booking data for parliamentary events and more.

We also consulted non-governmental/parliamentary sources, including UK and global media, and Tobacco Tactics, the TCRG’s knowledge exchange platform. Where information was not available, Freedom of Information (FOI) requests were used to identify additional incidents and further investigate others.

The report also underwent a process of external consultation. We consulted several of the UK’s leading public health and tobacco control experts to identify additional incidents and shared the draft report for validation by civil society organizations and UK government representatives. We shared early drafts of the UKTI with GGTC for technical input on relevance of the evidence to the implementation of the Article 5.3 guidelines. We also sought legal advice where we were unsure whether incidents were within the scope of the report, and/or were violations of Article 5.3.

Tobacco industry monitoring and research requires considerable time and energy. The report was authored by a dedicated team of researchers, supported by external partners. While most of this work took place during spring and summer 2023, there were several important tasks which took place following submission of report: policy engagement with civil society organizations and with the Department for Health and Social Care, as well as production of a Policy Brief with specific recommendations for government.

Finally, our industry monitoring is ongoing, and any potential violations of Article 5.3 are documented for assessment and possible inclusion in the next edition of the Index.

## PRACTICAL EXAMPLES

### Lobbying registers

There are two main lobbying registers in the UK: the Register of Consultant Lobbyists^6^, and the Scottish Parliament Lobbying Register^7^.

The former is UK-wide, but applies only to professional lobbyists who are VAT registered. The Scottish Parliament Lobbying Register is more comprehensive, requiring declaration of any face-to-face meetings (in person or via video conference) with Members of the Scottish Parliament (MSPs) and other public officials in Scotland for which the lobbyist was paid^8^. While we found no instances of tobacco industry lobbying on the Register of Consultant Lobbyists, multiple incidents from the Scottish Parliament Lobbying Register were included. Many of these meetings were brokered by communications or public relations (PR) firms. We also recorded lobbying not only by or on behalf of tobacco companies, but also by third-party organizations with tobacco industry links^9^.

### Public consultations

The UK government runs public consultations to gather feedback on proposed policies and regulations, to which the tobacco industry may contribute. During the timeframe under analysis we identified six relevant consultations to which the tobacco industry had contributed, as well as the Khan review, a major review of the government’s tobacco control policies commissioned by the Secretary of State for Health and Social Care, which aimed to determine whether the government would achieve its ambition of making England smoke-free by 2030^10^.

We consulted all information available on government websites regarding these consultations, including (if available) any responses from both the tobacco industry and industry-linked organizations. We also checked the outcomes of the consultations to identify any potential influence. From the approaches used by the devolved nations of the UK, we identified best practice and issued recommendations to government.

### MPs’ and Lords’ registers of interests

The registers of interests are MPs’ and Lords’ declare interests such as property ownership, employment, shareholdings and the receipt of gifts and hospitality. The registers of MPs’ financial interests are updated every two weeks when parliament is sitting, and once a month at other times^11^. There are additional registers for ministers and parliamentary staff^12^. We searched these registers systematically in the data-gathering phase.

In the UK there is no legislation to prevent the tobacco industry from donating to parties, candidates or campaigns, and there are no limits on how much money any donor can give to a party or MP. However, during the timeframe of the report, political parties were required to declare any donation above £7500 (in 2024 this increased to £11180) and MPs any donation more than £500^13-15^.

### Industry sources

We also consulted a wide range of sources from the tobacco industry or third-party organizations which may work to represent its interests. These included retail associations with tobacco industry members, public relations and lobbying firms contracted by the tobacco industry or its associates, and think tanks with current or historic links to the tobacco industry^9^. We identified industry-funded research published by third parties, and websites for campaigns produced by the tobacco industry or its associates^9^. Industry websites and press releases were also of use, particularly for investigating corporate social responsibility (CSR) and other public-facing activities. This includes websites for specific CSR initiatives^9^. We also searched tobacco industry publications (including annual reports and reports from AGMs) and industry and retail media.

### Cabinet members and political party events

We investigated the activities, interests and associations of cabinet members who were in post during the survey period.

We also looked at websites and programs for specific events sponsored by industry or at which industry figures gave speeches. These included political party conference programs, which have contained events involving the tobacco industry or its associates, usually ‘fringe’ events (events separate from, but associated with, the main party conference)^9^.

### External media

Relevant media sources were identified through our regular monitoring, including the mainstream UK and international news media, and some independent media. We searched these publications for incidents reported within our timeframe.

We produced tailored Google Alerts, identifying key search terms based on the tobacco companies operating in the UK market, the products they sell, and the names of key industry players and third parties. We also ran advanced Google searches restricted to the period of investigation.

Additional information shared with us by colleagues and external collaborators was investigated. We consulted external partners including public health and tobacco control experts and included known incidents that related to health policy or were violations of Article 5.3.

### Government websites

We identified incidents on the websites of various government departments and agencies, primarily the Department for Health and Social Care, but also the Department for Environment, Food and Rural Affairs; the Department for Business, Energy and Industrial Strategy; the Department for International Trade; Trading Standards; Her Majesty’s Revenue and Customs (HMRC); and the Medicines and Healthcare products Regulatory Agency (MHRA). We searched for tobacco companies, known industry-linked actors and representatives of consultancies working with industry.

The resources we consulted included public consultations, policy reviews, press releases, calls for evidence, records of meetings, official letters, ministerial meeting agendas, ministerial hospitality records, HMRC records of its interactions with tobacco industry stakeholders, Trading Standards’ list of annual outputs for tackling tobacco smuggling, and others. We also consulted the website of government agencies (which are not part of government departments), for example when we identified potential conflicts of interest.

### Freedom of Information requests

Requests submitted to public authorities under the UK Freedom of Information (FOI) Act (2000) can be a powerful tool for accessing information which has not been made publicly available^16^, and to explore corporate activity and potential policy influence^17^. While FOI requests have been used previously to expose corporate tactics and specific interactions between government representatives and the tobacco industry^18,19^, this is the first time they have been used systematically to gather evidence for the UKTI.

In February 2022, we submitted 973 FOI requests using the paid for version of a dedicated online tool developed by a UK charity. This enabled the submission of multiple requests at the same time and automatic tracking of responses. Owing to technical limitations, we were not able to create bespoke lists of organizations to target, and therefore used two pre-generated lists. Requests were sent to 339 local authorities (city, county and district councils and unitary authorities), making up 41% of submissions. The other list (n=574; 59%) was very broad. As well as all government ministries and departments, it covered a wide range of other state-run or state-funded entities. These included advisory committees, research councils, legal services, cultural bodies, and some state-owned companies.

Nearly all local authorities (93%) responded within the timeframe. However, to prevent the automated system from repeatedly resending duplicate and erroneous requests, we withdrew a total of 262 (27%), most of which were on the second list of other organizations. Non-responses were calculated up to April 2022. Owing to the limitations of the second list, and these withdrawals, it was not possible to calculate an accurate overall response rate.

### Diplomats

We are aware from our previous research of the long history of UK diplomats supporting the tobacco industry (particularly British American Tobacco) in its business overseas^19,20^. While there is a requirement for overseas posts to record details of interactions with industry^21^, this information is not recorded systematically or routinely made publicly available. These incidents are harder to investigate as they take place outside the UK, and FOI requests are a valuable tool to further investigate potential violations of Article 5.3 picked up in local media sources or by personal communication. Sometimes additional admissions of interactions are revealed which have not been previously reported^19^. However, a major limitation is that FOIs can only be submitted to the central government department, the Foreign, Commonwealth and Development Office (FCDO) in the UK. They also have to be focused on only one of two embassies or consulates at a time, otherwise they are likely to be rejected for time and cost reasons. Nevertheless, some FOIs were submitted to investigate specific embassies when public information indicated possible interaction between UK diplomats and the tobacco industry.

## DISCUSSION

The work of the Tobacco Control Research Group (TCRG) is underpinned by Article 5.3 of the WHO FCTC, the guidelines of which (adopted at the third Conference of the Parties) detail the principles, recommendations, and measures necessary to protect public health policies from tobacco industry interference. They also refer to a role for civil society, non-governmental and non-state actors in monitoring the tobacco industry^22^. As such, TCRG is well placed to deliver the UKTI.

Our work is also informed by our Tobacco Industry Monitoring, Research and Accountability (TIMRA) model^23,24^, which we applied to the process of data gathering and investigative research used in production of the UKTI 2023. We were also guided by the SEATCA methodology defined in the GTI and the work of other in-country researchers producing their own indexes elsewhere in the world. Every edition of the GTI has reinforced the finding that the industry’s access to policy makers undermines public health, and needs to be monitored, identified, exposed and countered in order to make progress in tobacco control^25^.

In the UK, the government makes a vast range of data available to the public, including registers of interest, minutes of selected meetings, catering and hospitality registers, ministerial meeting agendas, etc. However, this information is often not gathered systematically or presented consistently. We also encountered some differences in approach between the UK administration and the devolved administrations in Scotland, Wales, and Northern Ireland, complicating matters further.

For example, the scope of the Register of Consultant Lobbyists is extremely narrow. It covers only lobbying by professional lobbyists, but not by lobbyists working in-house at companies. When the Register was introduced in 2015, the official responsible admitted that the law had been ‘very narrowly drafted’ and stated that a lobbyist working for a tobacco company would be under no obligation to register^26^. It also covers only meetings with ministers and permanent secretaries, but not special advisers or other more junior officials^27^. Transparency International estimates that less than 4% of all lobbying activity is captured by the Register^28^.

Though the Scottish Parliament Lobbying Register is far more comprehensive, it still has shortcomings. It requires declaration only of face-to-face meetings – but not of letters, emails, or other forms of interaction. It excludes interactions with certain types of junior official, such as an MSP’s members of staff^8^. There is also no requirement for the lobbyist to declare how much they have been paid to lobby. And while the lobbyist is required to input information on ‘Purpose of lobbying’, this is typically a brief summary and little information on what was said during the meetings is ever disclosed.

In the case of the parliamentary registers of interest, the information is not consistently presented; for example, sometimes MPs declare the full names of tobacco companies, whereas at other times just an abbreviation is given. Furthermore, with the registers being updated every two weeks during sitting periods and once a month at other times^11^, there is considerable overlap between the different documents, with single declarations appearing across multiple versions of the register. This makes searching the registers very time consuming and ultimately it can be hard to establish what an MP or Lord has declared in total over a given period of time. There have been calls from journalists and transparency researchers for greater consistency, and a digital register has been proposed^12^.

Similarly, the treatment of tobacco industry responses to public consultations has varied widely across time and across the different UK administrations. For example, in a 2022 review of regulations introduced in 2015–2016, tobacco industry responses were not identifiable, having been rolled into a single category (‘Business’) with the responses of other stakeholders^29,30^. In contrast, in the Khan review, responses from the tobacco industry and its associates were published^31^. This inconsistency, both in what information is available and in how it is presented, makes monitoring tobacco industry activity around these consultations particularly challenging. Indeed, in the Policy Brief accompanying the UKTI, one of our recommendations to government was that tobacco industry responses were consistently identified, published and then filtered out, to ensure that they have no bearing on any eventual policy development^32^.

FOI requests provided information about industry interactions which we would not have otherwise identified. There were a number of advantages to using an automated platform, including the provision of pre-formed lists of organizations, being able to submit and track multiple requests at the same time, and the availability of real time response rates. However, one significant disadvantage was the inability to distinguish automatically between responses stating that information was not held, as opposed to information not being collected or recorded. This is of particular relevance for the UKTI, as WHO FCTC transparency requirements mandate the collection and recording of interactions with the tobacco industry. The automatic resubmission of requests was both an advantage, given that it eliminated the need to resubmit large numbers of FOIs manually, but also a disadvantage, as it was not feasible to actively manage the resubmission process with so many submissions. This underlines the need to consider timescale as a limiting factor from the outset, and to adopt a more selective approach when choosing which FOIs to send.

Despite these limitations, the information we received was mostly additional to the data we had collected using other methods and will help inform our research processes in future. For example, an increased awareness of the number and type of government-related entities in the UK should enable a more targeted approach.

Overall, these methods enabled production of a thorough and comprehensive report, allowing us to identify where the UK government fell short of full implementation of Article 5.3 of the WHO FCTC, and make specific recommendations on how it could improve further. These recommendations were compiled into a Policy Brief which was shared with the media^32^.

However, like any report based on open-source intelligence, the UKTI 2023 is necessarily limited by the data which are publicly available. As discussed, while FOI requests were used to obtain data which might otherwise have remained hidden, further research might explore other techniques which may be used to access information which is not in the public domain.

Finally, compiling a detailed, national-level report on tobacco industry interference, covering a timeframe of two years, is a highly resource-intensive undertaking. Currently, the GTI brings together the respective indexes of countries around the world, to enable comparison and knowledge exchange. If this is to continue, teams working in low-resource settings around the world require access to sustainable resources and technical support.

## CONCLUSIONS

The UKTI 2023 is the product of many months of monitoring and investigation. Intelligence on tobacco industry interference was provided by the authors of the report, as well as external partners in the tobacco control and public health communities. The more research we carried out using the tools discussed above, the more relevant incidents we identified. This is part of the reason why the UKTI 2023 recorded a sharp increase in the score and the UK fell down the ranking in the GTI.

However, despite the depth and breadth of our research, the report is not exhaustive. Since the report was published, further incidents have been identified. Ultimately, given the limitations of the data available, it is impossible to guarantee that other violations of Article 5.3 of the WHO FCTC did not occur during the timeframe under analysis. The UKTI should therefore be understood as the baseline for tobacco industry interference in the UK, rather than the totality.

## Data Availability

Data sharing is not applicable to this article as no new data were created.
